# Host Richness Increases Tuberculosis Disease Risk in Game-Managed Areas

**DOI:** 10.3390/microorganisms7060182

**Published:** 2019-06-24

**Authors:** Jose Angel Barasona, Christian Gortázar, José de la Fuente, Joaquín Vicente

**Affiliations:** 1 Animal Health Department, VISAVET Centre, Universidad Complutense Madrid, Avenida Puerta de Hierro s/n, 28040 Madrid, Spain; 2 SaBio Group (Health and Biotechnology), Instituto de Investigación en Recursos Cinegéticos (IREC; CSIC-UCLM-JCCM), Ronda de Toledo s/n, 13071 Ciudad Real, Spain; christian.gortazar@uclm.es (C.G.); josedelafuente@yahoo.com (J.d.l.F.); joaquin.vicente@uclm.es (J.V.)

**Keywords:** bovine tuberculosis, community assemblage, dilution effect, host diversity, transmission competence

## Abstract

Current scientific debate addresses whether species richness in animal communities may negatively moderate pathogen transmission and disease outcome (dilution effect), or to the contrary, if disease emergence benefits from more diverse community assemblages (amplification effect). The result may not depend exclusively on patterns of host species biodiversity but may depend on the specific composition of reservoir hosts and vectors, and their ecology. Host–pathogen interactions have shaped variations in parasite virulence, transmissibility and specificity. In the same way the importance of factors related to host exposure or to life history trade-offs are expected to vary. In this study, we demonstrate that ungulate host species richness correlates with increased community competence to maintain and transmit pathogens of the *Mycobacterium tuberculosis* complex (MTC) in game-managed areas in Mediterranean Spain. Therefore, we should consider natural and artificial variations in life histories of pathogens and host communities to characterize the impact of biodiversity on the health of diverse assemblages of human and animal communities. Since most approaches assessing epidemiology and transmission of shared pathogens only involve single- or pair-species, further research is needed to better understand the infection dynamics from complete community assemblages, at least in chronic diseases such as tuberculosis and in non-natural animal communities.

## 1. Introduction

Changes in host species diversity have been described as important factors influencing transmission risk of infectious diseases. More diverse assemblages would support a greater fraction of low-competence hosts, and therefore biodiversity losses may have the potential to increase disease (“dilution effect”) [1,2]. However, the principle underlying this phenomenon remains unknown. Some studies have claimed that healthy ecosystems may actually be richer in parasite diversity and biodiversity, even on a global scale, with this being associated with increased risk of zoonotic pathogens [3,4,5]; while others argue that preserving intact ecosystems and their endemic biodiversity should generally reduce the prevalence of infectious diseases [6,7]. In order to establish whether disease emergence, maintenance and risk of transmission maybe determined by particular host community assemblages, specific but diverse examples are required [8,9].

The outcome of host richness changes for infectious disease risk depends on a community´s ability to support infection–community competence [1,2,3,4,5,6]. In this study, we aimed to establish the correlation between host species richness and the competence of the whole community [1,6] to maintain and transmit tuberculosis (TB) in managed scenarios from Mediterranean Spain. We used a multi-host pathogen system that is well suited to address questions involving assembly because it provided replicate assemblages and a gradient of host richness (here, 1 to 4 species). TB caused by members of the *Mycobacterium tuberculosis* complex (MTC) affects a wide range of susceptible mammal species [7,10]. TB infection presents particularities: it usually develops into chronic infections, with long-term persistence in populations and low induced immunity. MTC is therefore able to induce a period of infectiousness in which direct contact between individuals occurs, which may favour transmission by both direct and indirect contact. However, the role of wildlife species has often been evaluated in single-species studies, neglecting the effects of multiple hosts and community structure on the MTC dynamics [11,12]. Studies which adopt a community perspective are therefore needed to better understand the complex effect of the structure of livestock and wildlife populations on MTC transmission scenarios [7,13,14]. This study thus aims to demonstrate whether ungulate host species richness operates with increased or decreased community competence to maintain and transmit the MTC.

## 2. Materials and Methods

### 2.1. Field Surveys and Disease

The study was performed in 45 game estates and extensive cattle farms of south-western Spain (37°13′48″ N to 39°31′43″ N in latitude; 2°25′54″ W to 6°34′06″ W in longitude). This area has a high ungulate density, composed by Mediterranean forests/scrublands (mainly composed of oak trees *Quercus* spp.) interspersed with typical agroforestry systems called “dehesas” (savannah-like habitats, 3.1 million ha in Spain, composed of pastures that mainly include oak trees). Large extensions of dehesas have been devoted to big game hunting during the last few decades.

We used field surveys to quantify TB prevalence in ungulate hosts and to establish patterns of host species composition, assembly and species abundance. For sampling, we chose sites where a priori a range of host densities and assemblages would be present [15,16,17]. The ungulate species in the study communities that are susceptible to TB caused by MTC are Eurasian wild boar (*Sus scrofa*), red deer (*Cervus elaphus*), fallow deer (*Dama dama*) and cattle. TB lesion prevalence confirmed by culture was used to estimate disease rates of each species (which is the common approach in wildlife/livestock TB studies, e.g., [15,16,18]). TB lesions were diagnosed by necropsy of the entire animal with detailed macroscopic inspection of lymphodes and thoracic organs [15]. At least one experienced wildlife pathologist (Drs. Vicente and Gortazar) supervised the necropsy, sampling and examination of tissues done by qualified veterinarians that had extensive experience in the diagnosis of macroscopic TB-compatible lesions. This examination routinely included retropharyngeal, submandibular, tracheobronchial and mediastinal lymph nodes and the entire lungs. Gross lesions in other locations were also recorded. Cultures using pyruvate-enriched Löwenstein–Jensen medium were performed to confirm TB infection [19]. Presence of TB lung lesions and culture was used to measure species competence in 45 wild ungulate populations (1752 wild boar, 1910 red deer and 329 fallow deer) and extensive cattle populations (1309 individuals) in Southern Spain from season 2003/2004 to 2015/2016, with species richness ranging from 1 to 4 (Table S1).

### 2.2. Host Abundances

A hunting index of population for red deer, fallow deer and wild boar per sampling population was used to calculate relative abundances of hosts [19]. Hunting estate data were collected during hunting events from 2003 to 2016 and were also provided by the regional government. We used the average of the total annual hunting bags for red deer, fallow deer and wild boar as a proxy to a relative population abundance according to Acevedo et al. [20]. In addition, we validated this data with a relative abundance index previously obtained in wild boar populations [21]. In summary, this study modeled the wild boar relative abundance (animals hunted per 100 km^2^) on more than 6000 hunting estates. The model, which was validated beforehand using independent data, was then extrapolated to the rest of the regions. The cattle population size is well known and data were provided from livestock sanitary authorities.

### 2.3. Species and Community Competence

Transmission of TB requires the establishment of initial local TB lesions, and hosts manage to keep them latent, hardly shedding mycobacteria to the exterior at this stage. Realized transmission success requires subsequent dissemination and excretion of bacilli, leading to chronic disease with lung involvement, open lesions, abundant shedding of mycobacteria and mortality. Therefore, as a measure of host competence (the ability of a host species to maintain and transmit TB) we used the presence of a TB lung lesion confirmed by culture, linking infection to development of severe infectious TB. Host competence was empirically estimated for each sampling population as the percentage of individuals with pulmonary involvement of TB, e.g., at least with one TB confirmed lesion. Community competence for the ungulate was calculated as in Johnson et al. [1], for which we multiplied each species’ competence by its relative abundance. Briefly, community competence (*p*) was calculated as

p=∑i=1nciSi∑i=1nSi

where $c_{i}$ is the competence of species i and $S_{i}$ is its abundance (see above). Table S1 shows sample communities attributes as concerns MTC lung lesion prevalence, total population and relative abundance of ungulates, number of species, and MTC species competence and community competence.

### 2.4. Statistics

Linear models (LM) were used to model community competence (response variable) as a function of the number of host species per community (level 3 and 4 hosts were grouped due to low number of estates/farms in level 4, *n* = 5) and total abundance of hosts (as continuous factor). This statistical approach was chosen based on the nature of the data and type of inferences under consideration. The assumptions of normality, homogeneity and independence in the residuals were met. Spearman’s rank correlation was used to compare species competence values and specific-host abundance rates per study site. For species differences, the MTC competence were statistically compared using a Kruskal–Wallis test. All statistical tests were performed at a significance level of *α* = 0.05. Statistical analyses were performed using IBM SPSS Statistics 20 and SAS 9.0 statistical software (IBM, Armonk, NY, USA).

### 2.5. Ethics Statement

The Ethics Committee on Animal Experimentation and Biosafety Handling of the Castilla-La Mancha University approved the procedures in such research (ref. 35/2013), which were designed according to European (86/609) and Spanish laws (R.D. 223/1988, R.D. 1021/2005), and current guidelines for ethical use of animals in research [22].

## 3. Results

**Figure 1:**
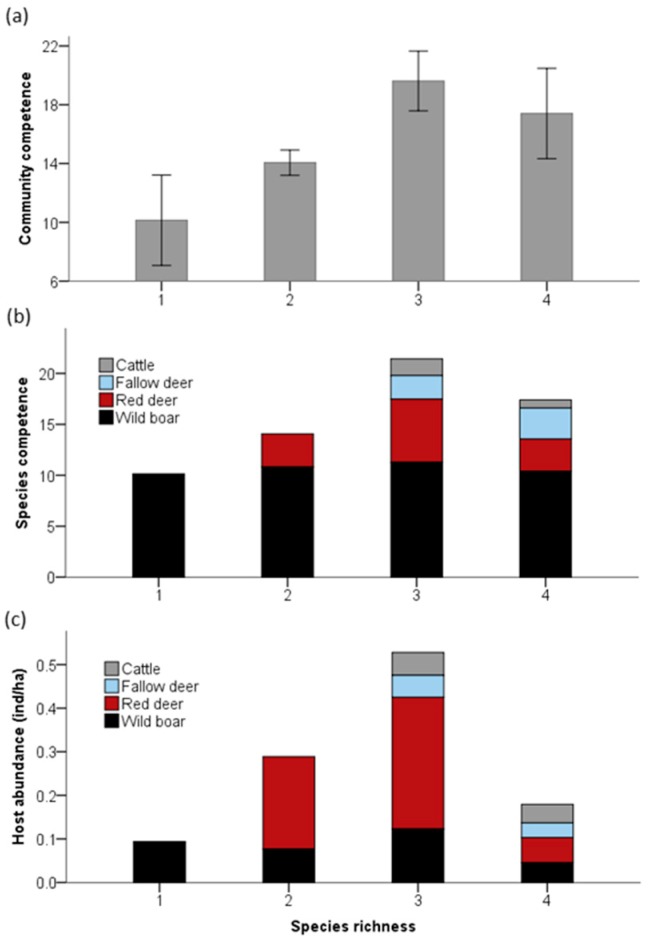
Influence of ungulate species richness on the community capacity to support tuberculosis infection. (**a**) Mean community competence as a function of species richness. Error bars represent 95% confidence intervals. (**b**) Species competence as a function of the host richness. (**c**) Host species abundance as a function of species richness.

We found a 46% increase in community competence for transmitting and maintaining TB (realized transmission) in richer assemblages with three or four species (average ± SE, 18.96 ± 9.63) compared with estates where only one species is present (10.29 ± 2.87; LM, f.d. = 2, F = 3.32, *p* = 0.046; Figure 1a). However, no effect in community competence was related to host abundance rates (LM, f.d. = 1, F = 1.349, *p* = 0.252).

**Figure 2:**
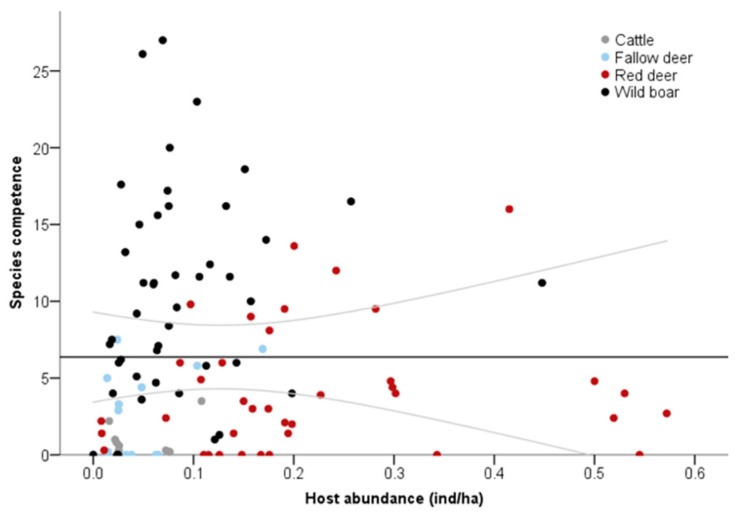
Comparison of ungulate host abundance and the species competence to support TB infection. Grey lines represent 95% confidence intervals.

Reservoir competence for TB varied widely among ungulate species (Kruskal–Wallis test, Z = 34.88, *p* < 0.001), remarking the higher values for wild boar all over the gradient of community richness (Figure 1b). The introduction of less frequent but still highly competent hosts in more rich assemblages increased host community competence for TB (Figure 1b). This was not attributable to changes in the abundance of the most competent hosts as infection increased without changes in general or specific host abundance (Spearman’s rank correlations, *p* > 0.05, Figure 1c and Figure 2).

## 4. Discussion

This study set out with the aim of assessing the importance of the host–pathogen interaction paradigm in a host rich community. While highlighting the benefit of a community-based approach to the study of infectious diseases [1], our findings provide evidence that increases in biodiversity do not necessarily reduce disease risk, at least in managed scenarios. Major factors distinguished some infectious agents from others [23] since life history affects parasite virulence, transmissibility, specificity as well as host defenses, and life strategies [24]. For example, a recent study [1] has provided seminal empirical and experimental evidence that amphibian species richness in natural communities negatively moderates the transmission and disease caused by the trematode, *Ribeiroia ondatrae*, which could be explained by the fact that defenses are costly and incur in trade-offs with resource investment.

However, many of the infection patterns of microparasites such as mycobacteria causing TB are determined by their ability to directly multiply in in the host, regardless of the host life history traits. A possible explanation is that a mass-action mode of transmission would benefit from increased niches offered by higher host diversity, resulting also in increased and more diverse networks of direct and indirect contacts (trophic relationships and host aggregation) [19], preventing the dilution effect. This evidence applies in particular to multi-host pathogens such as mycobacteria capable of persisting in the environment [25,26]. Most infections with these pathogens increase fitness costs only at very advanced stages, thus posing no important risk for species extinction in the community during most of their life span.

While existing studies have provided excellent scientifically supported examples with contrasting results, they often refer to very specific host communities and parasite assemblages. However, the TB case examined in this study demonstrates how a pathogen can interact in different ways with host communities, since parasite transmission and persistence is a complex interplay between hosts and parasites [14,27]. Furthermore, our findings contrast with similar approaches in Africa where the incompetent host species for MTC transmission are more likely to be present in high-diversity communities rather than in low-diversity communities [7,14]. Results from different epidemiological scenarios confer an additional value to the study of multi-host pathogens in wildlife populations. Although communities´ assembled richness is desirable for ecosystems function and conservation, its role in controlling infectious diseases remains debatable. It is also necessary to provide a deeper understanding of the epidemiology, because the plethora of associated pathogens may respond differently to changes in biodiversity.

In sum, if there is an argument to be made for redirecting scarce public health or conservation resources, it is critical to understand whether the relationship between biodiversity and disease risk is as general as has been suggested. However, we are unaware of any formal assessment of the generality of the dilution effect [28]. If there is no straight forward relationship between biodiversity and risk of zoonotic disease, then integrated approaches to disease control may require a more detailed understanding of the transmission ecology of a specific pathogen, vector and host species.

## Supplementary Materials

The following are available online at https://www.mdpi.com/2076-2607/7/6/182/s1, Table S1: Sample communities attributes: *Mycobacterium tuberculosis* complex (MTC) lung lesion prevalence as a measure of realized MTC, infection, total population and relative abundance of ungulates, numbers of species, and MTC species competence and community competence. n is indicated.
